# Evaluation of the association between gastric cancer and plasma selenium in Zambian adults: a case–control study

**DOI:** 10.3332/ecancer.2022.1351

**Published:** 2022-01-27

**Authors:** Kanekwa Zyambo, Paul Kelly, Violet Kayamba

**Affiliations:** 1 Tropical Gastroenterology and Nutrition Group, University of Zambia School of Medicine, PO Box 50398, Lusaka 10101, Zambia; 2 Blizard Institute, Barts & The London School of Medicine and Dentistry, Queen Mary University of London, 4 Newark Street, London E1 2AT, UK

**Keywords:** gastric cancer, atrophy, intestinal metaplasia, Selenium

## Abstract

There is some evidence that Selenium (Se) is protective against gastric carcinogenesis, but these data are inconsistent. With a predicted increase in gastric cancer cases in Africa over the next 20 years, there is an urgent need to identify strategies that could be employed to prevent the surge. The objective of our study was to investigate the association between gastric cancer and plasma Se levels in Zambian adults. Our method used a case–control study with cases having either confirmed gastric cancer or premalignancies and controls having none. In addition, we measured antibodies against Helicobacter pylori and human immunodeficiency virus. Data were analysed with Stata 15 software using standard statistical methods. Using a normal reference range for Se of 0.9–1.9 μmol/L, 140/159 (88%) study participants had Se deficiency. Plasma Se levels were similar in all the three groups; 0.33 (interquartile range (IQR) 0.14–0.64) μmol/L for patients with gastric cancer, 0.38 (IQR 0.21–0.60) μmol/L for premalignant lesions and 0.28 (IQR 0.14–0.64) μmol/L in controls, (*p*-values = 0.35 and 0.34, respectively). In conclusion, we found no association between plasma Se levels and gastric cancer or premalignant lesions among adult Zambian adults.

## Introduction

Selenium (Se) is an essential micronutrient which exerts its biological activity through selenoproteins [1]. It is a potent antioxidant and anti-inflammatory agent and has therefore been investigated in several disease conditions, including cancer [2, 3]. However, excessive amounts of Se have also been shown to have detrimental health effects [4].

Gastric cancer is one of the leading causes of cancer-related mortality worldwide. In 2020, there were over a million cases of gastric cancer, with 768,793 related deaths [5]. It is estimated that by 2040 cases of gastric cancer will have increased by over 60% [5]. The role of micronutrients in gastric cancer development has been evaluated by several investigators with inconsistent conclusions [6]. With its known antioxidant properties, Se is thought to be protective against gastric cancer [7, 8], and low levels have been reported in gastric cancer patients [9, 10]. Analysis from the NutriNet-Santé cohort showed that higher Se intake was associated with a decreased digestive cancer risk [11]. In addition, Se was also reported to have a role in reducing the effects of known carcinogens [12]. With continued advances, there also is evidence that Se could have a role in gastric cancer therapy [13, 14].

Diet is the major source of Se and availability in plant-based foods varies depending on the amounts present in the soil [4, 15]. Similarly, for animal products, such as milk and eggs, the levels depend on quantities in feeds used to raise the animals [4]. These factors contribute to observed population variations in Se levels in different parts of the world [4]. A recently conducted Cochrane review found no evidence that increasing Se intake through diet or supplementation prevented cancer in humans [16]. It is thought that incremental doses of Se would be beneficial against cancer [17], but a study evaluating tissue accumulation of Se showed that levels were higher in gastric cancer tissues than non-cancerous ones [18]. With this work on the influence of Se on carcinogenesis, it remains unclear what Se levels would be most beneficial for cancer prevention.

The burden of gastric cancer appears to be increasing in sub-Saharan Africa, but there are limited data implicating micronutrients such as Se in this predominately low resource region. Se status in southern/central Africa is known to be poor [15], so we took the opportunity of a recently completed case–control study of gastric cancer to test the hypothesis that gastric cancer may be related to Se deficiency.

## Methods

This was a case–control study conducted at the University Teaching Hospital in Lusaka, Zambia. It was part of a larger study evaluating risk factors for gastric cancer in a Zambian population [19]. The study participants were enrolled from among patients referred for esophagogastroduodenoscopy (OGD). All patients signed informed consent to participate. Cases were patients either with histologically confirmed gastric adenocarcinoma (gastric cancer) or premalignant lesions (atrophy, intestinal metaplasia or dysplasia). Controls were patients who presented for OGD but were found to have no gastric cancer or premalignant lesions. Patients with a prior history of gastric cancer treatment were excluded from the study. The study was approved by the University of Zambia Biomedical Research Ethics committee, reference number 000-03-16.

### Study procedure

OGD was carried out on the patients under light sedation as previously described [19]. At least six biopsies were taken from lesions suspected of being malignant. Biopsies were also obtained from patients without any visible lesions: two each from the antrum, incisura and body along the greater curvature. This was done to check for the presence of gastric premalignant lesions. Biopsies were immediately placed in buffered formalin-saline and sent for histopathology. A technician processed these biopsies using standard methods and histological diagnoses were carried out by an experienced pathologist. Peripheral blood (plasma later extracted) was collected and stored at −80°C for further analysis of Se levels. Basic clinical characteristics were obtained using interviewer-administered questionnaires. The month of patient recruitment was recorded and categorised into the three seasons in Zambia: cold and dry (April–July), hot and dry (August–October) and hot and wet (November–March).

### Measurement of plasma Se

Plasma Se levels were measured using the Optima DV 7000 (Perkin Elmer, Midrand, South Africa). Following collection, blood was centrifuged at 856 g for 15 minutes at 4°C to extract plasma, then stored in a −80°C freezer. Three triplicates were carried out per sample. Diluent containing 2% nitric acid, 3 M HCl and 1% Triton was used to digest the sample. To create a standard curve, five working standard solutions ranging from 0.5 to 2.5 μmol/L (PerkinElmer Pure Instrument Calibration Standard 4-N930021) were used. Seronorm L-1 (SERO AS 0903106) 1:9 v/v with diluent and a pooled plasma sample were used as quality control which was rerun after every ten samples. Plasma samples were diluted (1:9 v/v) with a diluent. Instrument working conditions were set at wavelength 196.026 nm, plasma gas flow 1.5 L/minute, auxiliary gas flow 0.2 L/minute, nebuliser gas flow 0.6 L/minute, RF Power 1450 W and peristaltic pump flow rate 1.8 mL/minute.

### Measurement of antibodies against Helicobacter pylori (HP) and human immunodeficiency virus (HIV)

Testing for HIV antibodies was done using Uni-Gold™ rapid diagnostic kits (Trinity Biotech, Wicklow, Ireland). IgG antibodies against HP were quantitatively measured in U/mL with enzyme-linked immunosorbent assay by Abcam (ab108736), Cambridge, UK.

### Statistical analysis and sample size estimation

We powered the study to determine a difference between gastric cancer cases and controls. We used proportions from an Iranian study in which they reported Se levels of 111.6 ± 27.7 μg/L in gastric cancer patients and 129.9 ± 32.1 μg/L in controls [10]. With an 80% power and alpha of 0.05, we needed at least 44 patients in each group.

All continuous variables were summarised using medians and interquartile ranges as they were non-parametric, and proportions were computed for categorical data. Normality was tested using the Shapiro–Wilk test.

Two-way analyses were employed to look for associations between gastric cancer or premalignant lesions and exposures of interest using the Kruskal–Wallis test for multiple values and Fisher’s exact test for binary variables. Confidence intervals at 95% were used with *p*-values less than 0.05 being considered statistically significant. A stepwise logistic regression analysis was carried out to evaluate associations while adjusting for possible confounders. Data were analysed using STATA version 15 (College Station, TX, USA).

## Results

Over 21 months, we enrolled 159 patients. Among them, 56 (35%) had gastric cancer and 18 (11%) had gastric premalignant lesions. These patients were compared to 85 (54%) controls. Of those with premalignant lesions, 6/18 (32%) had atrophy without intestinal metaplasia and 12/18 (68%) had atrophy with intestinal metaplasia. None of the patients had dysplasia.

The median age was 62 years (IQR = 52–72 years) for gastric cancer patients, 56 years (IQR = 40–69 years) for those with premalignant lesions and 56 years (IQR = 41–60 years) for the controls. There was a statistically significant difference in age between controls and patients with gastric cancer (*p* < 0.001) but not premalignant lesions (*p* = 0.33; Table 1). Similarly, gastric cancer patients had a significantly low body mass index (BMI). HIV-positive status was similar in all the groups at about 20% (Table 1).

### Plasma Se comparison among the three patient groups (gastric cancer, premalignant lesions and controls)

**Figure 1.**
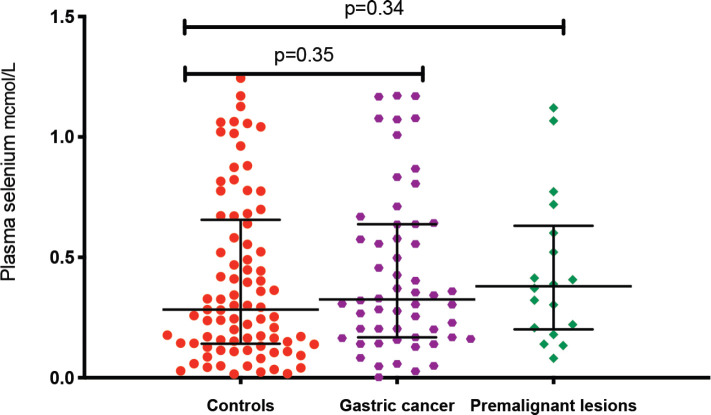
Plasma Se levels in the three patient groups: gastric cancer, premalignacy and controls. *Test for association computed using the Kruskal–Wallis test. **Error bars represent median with interquartile ranges.

**Figure 2.**
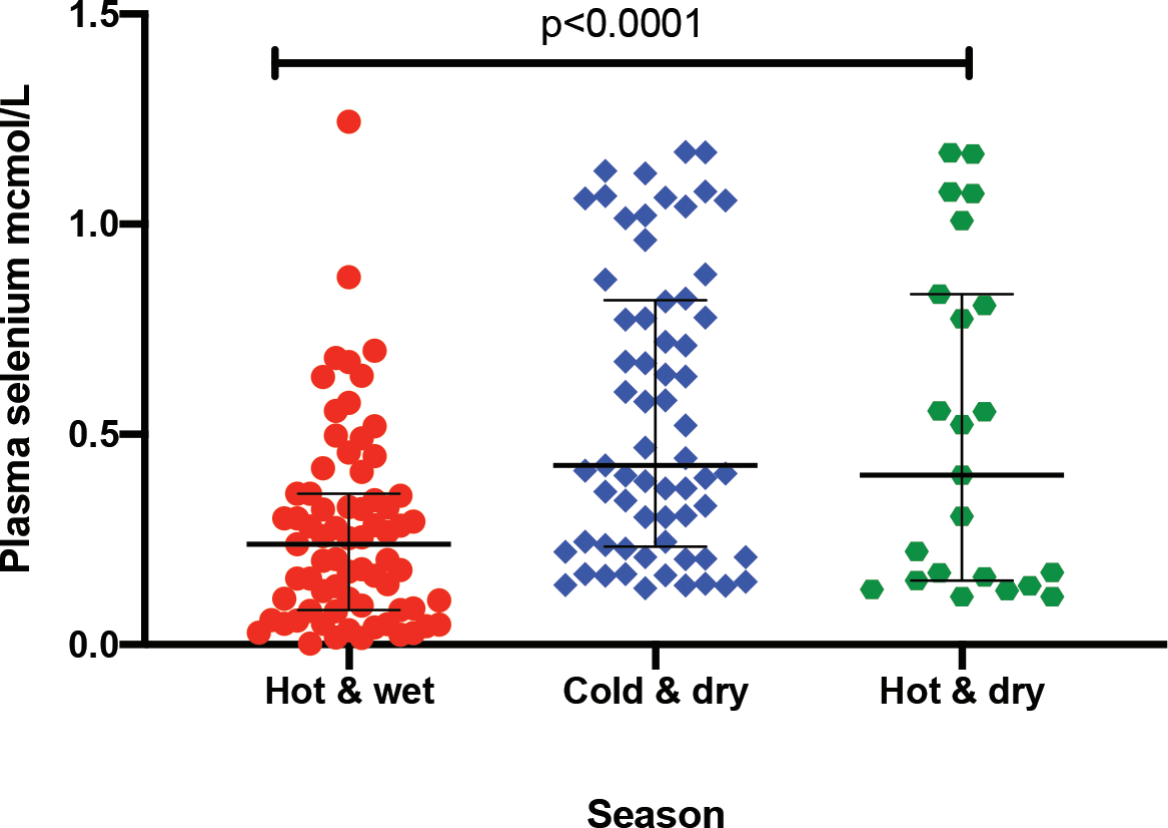
Plasma Se by season: hot and wet is from November to March; cold and dry is from April to July; and hot and dry is from August to October. *Test for association computed using the Kruskal–Wallis test. ** Error bars represent median with interquartile ranges.

The median plasma levels of Se were 0.33 (IQR = 0.14–0.64) μmol/L in gastric cancer patients, while it was 0.38 (IQR = 0.21–0.60) μmol/L in controls, a difference that was not statistically significant (*p* = 0.35). The median level in patients with premalignant lesions was 0.28 (IQR = 0.14–0.64) μmol/L, which similarly was not significantly different from the Se level in controls (*p* = 0.34; Figure 1). Using a normal reference range for Se of 0.9–1.9 μmol/L [20], 49/56 (88%) gastric cancer patients, 16/18 (89%) patients with premalignant lesions and 75/85 (88%) controls had low Se levels. Low serum Se was not associated with either gastric cancer (OR = 0.9; 95% CI = 0.3–3.1, *p* = 1.0) or premalignant lesions (OR = 1.1; 95% CI = 0.2–10.9, *p* = 1.0). Dividing the Se levels into four quartiles, there was no difference between the two groups of cases and controls (all *p*-values > 0.05; Table 2). The measured Se levels were lowest in the hot and dry seasons (Figure 2), but this had no influence on the above-mentioned comparisons. A stepwise logistic regression model, including the confounders age, sex, type of patient (gastric cancer or premalignant lesion and controls), season, BMI, HIV status and HP antibodies, did not show any association between gastric cancer or premalignant lesions and low Se levels (all *p*-values > 0.05). Separate models were employed for gastric cancer cases and those with premalignant lesions.

## Discussion

The role of Se in gastric carcinogenesis is not clear. In this study, we report a high proportion of low Se levels among Zambian adults with no association with gastric cancer or premalignant lesions.

It is known that micronutrient deficiencies are commonest in communities with poor dietary diversity [21], which includes many low-income African countries [15]. In Malawi, for example, a representative population of 2,761 people revealed Se deficiency prevalence of about 60% [22]. Another study, also from Malawi, estimated that more that 50% of the households were at risk of Se deficiency [23]. Gastric cancer, despite its predicted rise in cases over the next 20 years, is not among the most prevalent cancers in Africa [5], which strengthens the argument that Se deficiency might not necessarily be a major contributing aetiological factor. However, carrying out comprehensively designed high-quality studies on gastric cancer in a low resource setting is limited by scantly endoscopy services, poor expertise and a generally limited financial investment. Similarly, studying Se intake or estimating its status requires additional resource mobilisation. The main source of Se is dietary [24], but accurately predicting the status from oral intake is a challenging [25]. Measurement of Se can be done from plasma, erythrocytes, selenoproteins and glutathione peroxidase activity [26], and there is recent evidence that urine measurements can also give reliable population level estimates [27].

HP is a major risk factor for gastric cancer so we analysed its potential link to plasma Se levels. Our previous work did not demonstrate any association between serological measures of HP infection and gastric cancer or its virulence factor CagA [28] and here we explored the possibility of a synergistic role of Se and HP. The role of Se on HP virulence has not been defined, but there are reports of differential Se statuses in direct comparisons between inhabitants of high and low gastric cancer incidence regions [29]. Studies evaluating this subject did not find an association between HP and Se [30, 31].

Se plays a role in immune system function and consequently the progression of HIV disease [32]. Patients included in this study were selected from among those coming to the hospital, resulting in a higher prevalence than the national figures for Zambia. We found no association between HIV and Se levels in this study, but had also previously reported no link between gastric cancer and HIV infection [28]. Our finding of seasonal variations in Se levels could be a reflection of fluctuations in Se intake, which is largely dependent on consumed food. Generally, the measurement of plasma Se is an indicator of short-term status and would therefore be affected by seasonal dietary changes [33]. Using logistic regression, we were able to show that seasonal Se changes in our patients did not influence our overall conclusion.

To the authors’ knowledge, this is the first study evaluating the absence of link between gastric cancer and plasma Se in Zambia. However, the study has some limitations. Firstly, plasma measurements do not allow for long-term assessment of Se status, which is what would be most informative as carcinogenesis in not an acute process. Secondly, the study was powered to detect a difference for gastric cancer patients but not those with premalignant lesions. Therefore, we could have missed an effect with the latter group.

## Conclusion

In conclusion, there was a high proportion of low Se levels in the study participants. We found no association between plasma Se and gastric cancer or its premalignant lesions. It is, therefore, unlikely that Se is a driver for gastric carcinogenesis in this population.

## List of abbreviations

HIV: Human immunodeficiency virus
Se: Selenium

## Conflicts of interest

None of the authors have any conflicts of interest to declare.

## Funding

The research reported in this publication was partially supported by the Fogarty International Centre of the United States National Institutes of Health (NIH) under award number D43 TW009744 and the U.S Civilian Research and Development Foundation (CRDF Global) provided additional funding award number DAA3-16-62699-1. The content is solely the responsibility of the authors and does not necessarily represent the views of the NIH or CRDF Global.

## Figures and Tables

**Table 1.** Summary of the characteristics of enrolled patients in three groups: gastric cancer, gastric premalignancy and controls.

Characteristic	Controls *n* (%), *n* = 85	Gastric cancer *n* (%), *n* = 56	*p*-value	Controls *n* (%), *n* = 85	Gastric premalignancy *n* (%), *n* = 18	*p*-value
Sex Male Female	39 (46%) 46 (54%)	24 (43%) 32 (57%)	0.73	39 (46%) 46 (54%)	7 (39%) 11 (61%)	0.61
Age group (years) Less than 30 30–44 45–60 More than 60	3 (4%) 28 (33%) 31 (36%) 23 (27%)	1 (2%) 6 (11%) 18 (32%) 31 (55%)	<0.001	3 (4%) 28 (33%) 31 (36%) 23 (27%)	0 (0%) 7 (39%) 4 (22%) 7 (39%)	0.62
BMI (kg/m^2^); median (IQR)	24 (21%–27%)	18 (16%–21%)	<0.001	24 (21%–27%)	26 (23%–29%)	0.45
HP serology, U/mL	120 (92%–157%)	126 (95%–155%)	0.95	120 (92%–157%)	96 (88%–159%)	0.41
HIV antibodies Positive Negative	19 (23%) 65 (77%)	11 (21%) 42 (79%)	0.84	19 (23%) 65 (77%)	4 (22%) 14 (78%)	1.00

^a^The Kruskal–Wallis and Fisher’s exact tests were used for hypothesis testing (associations between various factors and gastric cancer or premalignant lesions)

**Table 2.** Evaluation of Se levels in gastric cancer, gastric premalignancy and controls divided into quartiles.

Se (μmol/L) levels in quartiles	Controls, median (IQR)	Gastric cancer, median (IQR)	*p*-value (comparison for gastric cancer and controls)	Gastric premalignancy, median (IQR)	*p*-value (comparison for premalignancy and controls)
First quartile	0.1 (0.04–0.1)	0.07 (0.05–0.2)	0.72	0.1 (0.08–0.1)	0.35
Second quartile	0.2 (0.2–0.3)	0.2 (0.2–0.3)	0.61	0.2 (0.2–0.3)	0.94
Third quartile	0.4 (0.4–0.5)	0.4 (0.3–0.6)	1.00	0.4 (0.4–0.5)	0.82
Fourth quartile	0.9 (0.8–1.1)	0.9 (0.7–1.1)	0.70	0.9 (0.7–1.1)	0.78

^a^The Kruskal–Wallis test was used for hypothesis testing (associations between Se levels in four quartiles and gastric cancer or premalignant lesions)
